# A New Simple Screening Tool—4QT: Can It Identify Those with Swallowing Problems? A Pilot Study

**DOI:** 10.3390/geriatrics5010011

**Published:** 2020-02-27

**Authors:** Karwai Tsang, Esther SY Lau, Mariyam Shazra, Ruth Eyres, Dharinee Hansjee, David G Smithard

**Affiliations:** 1 St Thomas’ Hospital, Lambeth Palace Road, London SE1 7EH, UK; karwai.tsang@nhs.net; 2 Royal Alexandra Hospital, Paisley, Renfrewshire PA2 9PJ, UK; esther.lau@nhs.net; 3 Northwick Park Hospital, Watford Road, Harrow HA1 3UJ, UK; mshazra@nhs.net; 4 Guy’s Hospital, St Thomas’ Road, London SE1 9RS, UK; ruth.eyres@nhs.net; 5 Queen Elizabeth Hospital, Stadium Road, Woolwich SE18 4QH, UK; dharinee.hansjee@nhs.net; 6 Dept Sports Science, University of Greenwich, London SE9 2UG, UK

**Keywords:** swallow screen, dysphagia, 4QT, frail

## Abstract

As people and the population age, the prevalence of swallowing problems (dysphagia) increases. The screening for dysphagia is considered good practice in stroke care, yet is not routinely undertaken in the management of frail older adults. A short swallow screen, the 4QT, was developed following a review of the literature. The screen has four questions relating to swallowing that can be asked by a member of the health care team. A convenience sample of 48 older frail patients on an acute frailty ward was recruited into a Quality Improvement project. Their swallow was screened using the EAT-10 and 4QT. A speech and language therapist assessed for the presence of dysphagia using a standardised assessment for dysphagia. The 4QT was as effective as the EAT-10 in identifying older frail adults with potential swallowing problems (Κ = 0.73). The 4QT has 100% sensitivity, 80.4% specificity and positive predictive value (PPV) 50%, negative predictive value (NPV) 100%. The 4QT is a highly sensitive but not specific swallow screen, only 50% of people reporting swallowing problems were confirmed to have a degree of dysphagia by the SLT. The 4QT is a simple screening tool that could be used by all staff, but requires further research/evaluation before it is widely accepted into clinical practice.

## 1. Introduction

Swallowing is a complex process [1] involving a modulated neuromuscular reflex. Swallowing may be affected by many different disease processes and consequently dysphagia (defined as difficulty with the passage of the bolus from the mouth to the stomach); oropharyngeal dysphagia (not including the oesophageal phase of swallowing) has been accepted as a geriatric syndrome [2,3,4,5].

Society is ageing due to more people surviving into adulthood and subsequently living longer. The older population is increasing and in many western populations >15% of the population are >65 years of age [6], Japan is particularly affected with an older population (>65 years) of 27% [6]. The number of very old people (>85 years) is showing the largest increase in percentage growth, 5% of the European population is >85 years of age [7]. Thirty percent (1.5% of the European population) of very old people are frail (vulnerable or older people with health-related disability, dependency and who have little or no physiological reserve) [5,8]. Frail older people often have complex, multiple long-term conditions and frequently require multiple medications which may be dose and time dependent [9]. 

The presence of dysphagia in the older population will vary depending upon the social/clinical setting and any underlying comorbidity [10]. Of older people living alone in the community, 15% are reported to have dysphagia and this prevalence increases with age, presence of frailty, dementia, stroke, and temporarily in acute illness [10]. Carrion et al. found that 47.4% of older people ≥70 years admitted to an acute geriatric unit in Spain had oropharyngeal dysphagia on assessment [11]. 

It is accepted that dysphagia is common in acute stroke (28–72%) [12], and that swallow screening/assessment is mandatory in many countries on presentation [13,14]. Bray et al. reported UK data from the Sentinel Stroke National Audit Programme (SNAPP), showing that a delay in assessment/screening by a SLT was associated with increased mortality and morbidity [15]. The prevalence of dysphagia during acute illness occurs at the same frequency in older frail people as in acute stroke [11], yet swallow screening is not routine [16]. 

The management of dysphagia, in an acute hospital setting, is a multidisciplinary and multistage process. The first steps are to identify those people with swallowing problems. This entails screening people soon after admission to hospital, prior to further assessment and treatment/management. Within stroke services, nursing staff are trained to screen/assess the ability to swallow using a water swallow test at the bedside [17] and if there are concerns, referral to a speech and language therapist for further assessments should occur. It has been difficult to implement a similar pathway on the acute frailty or acute medical wards. 

Referrals to speech and language therapy (SLT) services are increasing year on year resulting in increased pressure on the SLT service and they are unable to offer a routine screening service. There is, therefore, a need to empower frontline medical and nursing staff to screen prior to referral rather than assess peoples’ ability to swallow when they are on the ward. Previous work has suggested that medical and nursing staff are not confident to screen a person’s ability to swallow using a water swallow test [18], usually because they have not been trained to do so [18]. Therefore, a short and simple swallow screen is required, which can be used by medical and nursing staff to identify people who may have a difficulty swallowing (not an assessment to determine if aspiration is present) to assist in filtering referrals to the speech and language therapy service. 

The aim of this early phase study was to develop and evaluate a short swallow screen that could be used by “medical” staff prior to SLT referral for further assessment and management. 

As part of a Quality Improvement programme (QIP) to develop a simple bed side swallow screen to be employed by untrained health care staff, a two-stage approach was adopted.

## 2. Methods

### 2.1. Stage 1

The purpose was to determine those factors commonly used to identify an unsafe swallow that does not require the use of administering any liquids or foods. The reasoning behind this was to develop a swallow screen rather than a swallow assessment. Administration of liquids or foods in a swallow assessment would require training and a degree of competency/expertise as well as the availability of these elements out of hours.

#### 2.1.1. Methodology

A literature search was conducted using PubMed and Google Scholar and to identify papers that reported the development and validation of simple bedside assessments/swallow screens (e.g., Bed Side Assessment, EAT-10) [19,20,21,22,23,24] (Table 1). Search terms included “frailty”, “Swallow” “screen” “dysphagia” “old age” (The search of the literature was not undertaken in the manner of a formal systematic review; hence the PRISMA guidelines were not followed). The items contained within the swallow tool were listed (Table 2). 

#### 2.1.2. Results

From the literature, 30 different swallow screens were identified (using Pubmed, Medline and Google Scholar) (Table 1). 

One hundred and two different elements were identified from the swallow assessment/screening tools identified from the literature. Those elements that appeared most frequently are detailed in Table 2. The other elements were present on < 2 occasions. A total of 23 screens/assessments used a water trial. Finally, the items were ranked in order to identify those most frequently employed (Table 2). 

**Figure 1:**
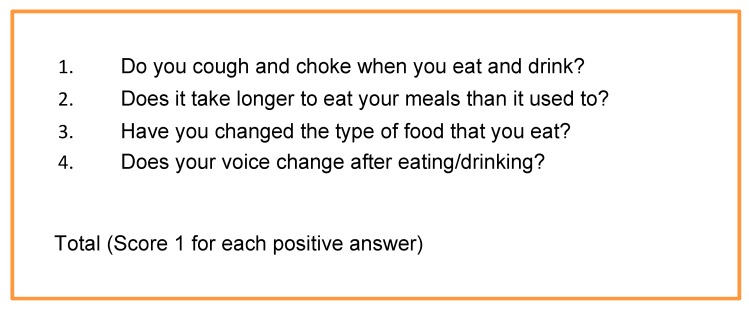
The 4QT.

Having identified these as common elements of swallow/screens assessments, we combined them in to a simple 4-point questionnaire test or 4QT (Figure 1).

A score of zero was taken to indicate that the participant had no swallowing problems (a similar approach was taken by Schrock et al. [24]), whereas a positive score on any of the elements was taken to indicate that there may be a problem with swallowing (but not the presence of aspiration).

### 2.2. Stage 2

Having decided to use the 4QT, the next phase was to evaluate its effectiveness as a swallow screen. The stage of the QIP was to assess whether medical staff were able to administer the 4QT, without training, and whether its sensitivity, specificity and predictive values suggested that the 4QT could be clinically useful.

Within our clinical service, referral to the SLT department is made if someone demonstrates difficulty when being observed eating and drinking. Therefore, the 4QT could be a way of proactively asking about difficulties when a clinical review is being undertaken rather than waiting for any observation. The two are complimentary and not mutually exclusive. 

#### Methodology

A convenience sample of 48 frail older people admitted to an acute Frailty Ward in an acute hospital in February and March 2018 was recruited. All those agreeing to take part provided verbal consent (written consent was not required as this work is part of a QIP).

Routine demographic data of age, sex and admission diagnosis were collected. All participants were assessed using the Clinical Frailty Score [25] (a score between 1 = not frail to 9 = terminal care) and cognition using the abbreviated mental test score (AMT: a categorical scale out of 10 as a screen for cognitive problems). These were collated to further describe the participants recruited.

Participants were identified for assessment when they were medically stable (not on the day of admission) and none had been referred to the SLT before agreeing to take part in the study.

**Figure 2:**
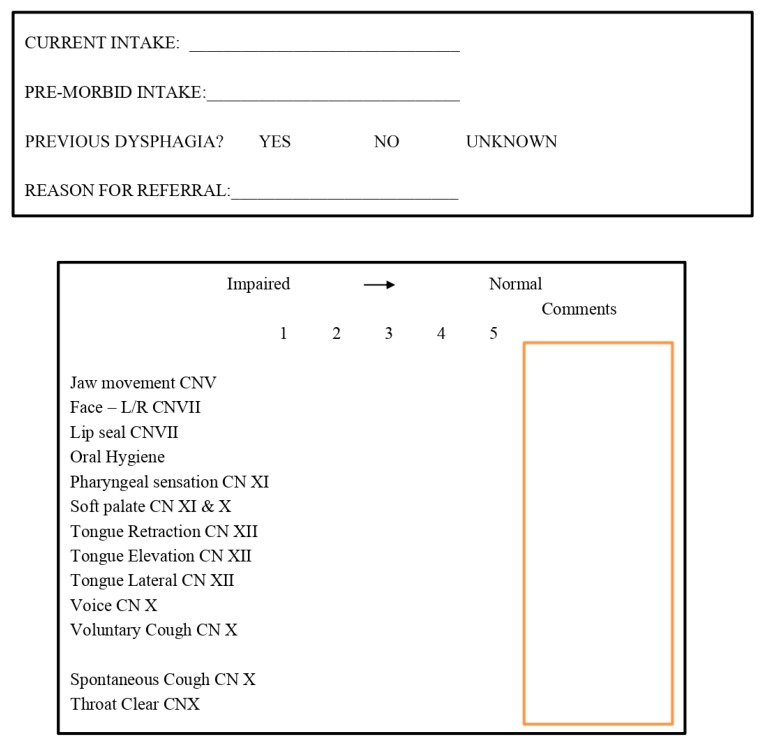

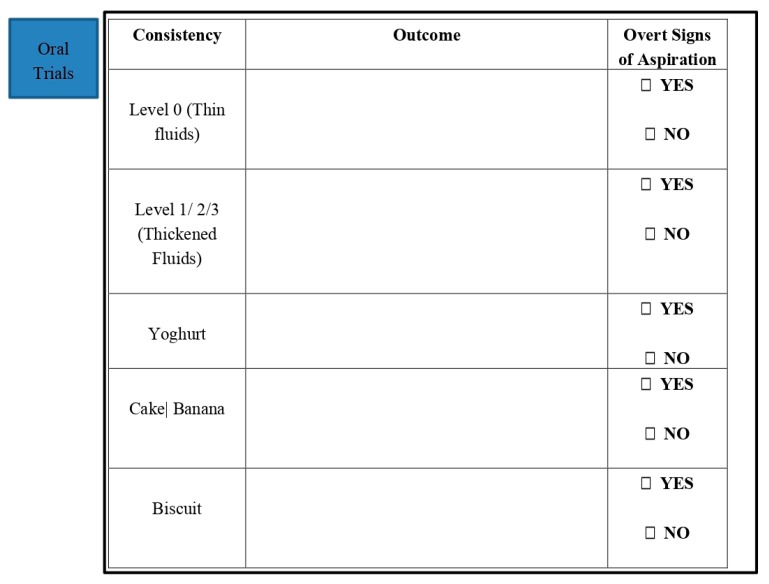
Speech and Language Therapy Assessment.

The usual clinical management pathway for all people considered to have difficulties swallowing is a referral to the SLT service. For the purpose of this pilot study, all participants were assessed by an SLT irrespective of the 4QT result. The SLTs used a standard departmental assessment tool, prior to any further instrumental assessment and intervention (Figure 2). All other assessments (4QT, CFS and EAT-10) were undertaken by a member of the medical team.

The SLT assessment was used as a clinical “gold standard” for the presence of dysphagia. Imaging was not used as the purpose of the 4QT is not to detect aspiration. The outcome of the SLT swallow assessment was either no dysphagia evident, or dysphagia evident with further management plans instigated.

It was considered appropriate to compare the 4QT to another short questionnaire, and at the time the EAT-10 [19] was the most appropriate tool available. Although it is usually self-administered it has been administered by staff in certain care home settings [26]. The cut off value used was that of 3+ as per the original papers [19]. Each person had their swallow screened using the 4QT, EAT-10 and a bedside assessment conducted by a SLT. 

Results were analysed to assess the specificity, sensitivity, positive predictive value and negative predictive value against the safe/unsafe result of the clinical SLT screen.

## 3. Results

The median age of those taking part was 83 years (range 75–102). A total of 58% (28) were male. The median clinical frailty score was 4 (range 1–7); 30 (62.5%) had a clinical frailty score of 5 or less. The inpatient diagnosis was diverse but representative of an acute frailty ward (Figure 2).

**Figure 3:**
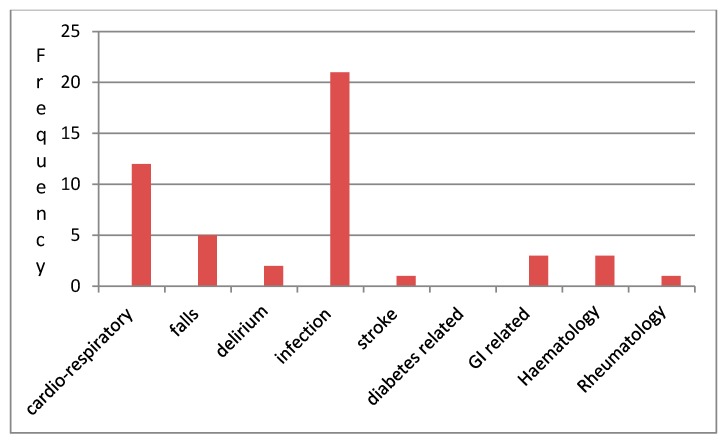
Admission diagnoses.

Cognitively, the mean AMT on admission was 8/10. Only 4 (8.3%) people had a prior history of dysphagia and only eight (16.7%) had any mention of their safety to swallow documented in the medical record. The primary diagnosis was variable (Figure 3), but none had presented with an acute stroke. 

On screening, using the 4QT, 14 (29%) were considered to have swallowing problems, and 22 (45%) with the EAT 10. The SLT assessment identified only seven participants to be unsafe. The agreement between the EAT-10 and 4QT using the Kappa score (a statistical measure of agreement: 0 = no agreement and 1 = perfect agreement) was 0.73 with a correlation of r = 0.673. The 4QT had a100% sensitivity and 80.4% specificity against the clinical gold standard of the SLT assessment, but like many other screens, its PPV was only 50% but the NPV was 100%.

## 4. Discussion

Dysphagia is not uncommon in the population as a whole: Chen et al. (2009), asked 120 community-dwelling older people whether they had swallowing difficulties, and 15% responded affirmatively [4]. Wilkins et al. (2007) reported a prevalence of 22.6% in an unselected primary care population [3]. Bloem et al. (1990) found that 16% of people >87 years responding to a questionnaire reported dysphagia [27]. In an acute setting the prevalence is even higher; Carrion et al. found that 47.4% of older people ≥70 years admitted to an acute geriatric unit in Spain had oropharyngeal dysphagia on clinical assessment using the V-VST [11].

Dysphagia is independently associated with increased morbidity and mortality [3,11] especially if comorbidities such as stroke are present. There are many components, both medical and social, contributing to this; if someone cannot swallow safely then they are at risk of malnutrition, choking and possible infection [28]. Swallowing problems are reported to be high in ill, older frail people [28], and as there is frequently failure to screen for them [16], many cases will not be identified resulting in increased morbidity [16,29].

Screening is defined, by the WHO as the presumptive identification of unrecognized disease in an apparently healthy, asymptomatic population by means of tests, examinations or other procedures that can be applied rapidly and easily to the target population (https://www.who.int/cancer/prevention/diagnosis-screening/en/). Wikipedia states that “Screening, in medicine, is a strategy used to look for as-yet-unrecognised conditions or risk markers in individuals without signs or symptoms. This testing can be applied to individuals or to a whole population. The defining features of screening programmes are that the people tested do not have signs or symptoms and the implied agreement is future risk reduction from an undesirable disease outcome” (Wikipedia accessed November 2019).

This paper reports the use of a screening tool (4QT) for oropharyngeal dysphagia in older frail people (not acute stroke), where the presence of swallowing problems is frequently unknown or unreported. The purpose of the tool is to identify/highlight those older frail people who may have a difficulty swallowing; its purpose is not to identify the nature or aetiology of any dysphagia present. Many studies report the use of a swallow screen; however, in reality, many are assessments. This is borne out by the systematic review of 1012 articles, conducted by Etges et al. (2014), where only 20 studies were selected, many of which were more akin to assessments rather than swallow screens [20].

The EAT-10 was used as a questionnaire-based standard screen/tool. The results show a high level of agreement between the EAT-10 and 4QT. The EAT-10 was initially developed to be a patient completed tool, but it has subsequently been administered by carers and over the telephone. At the time of this study, the research team considered the ET-10 the most appropriate tool to use as a gold standard/control screen.

A swallow screen needs to be simple to use and have a reasonably high sensitivity and specificity. The 4QT has a very high sensitivity but the specificity was only 80.4% which reflects in the poor PPV. This is similar to some other screens which use a timed water swallow as part of their screen. 

The use of a simple questionnaire-based swallow screen is not new and two other studies have reported similar results with similar screening tools. Uhm et al. (2019) reported a 12-point questionnaire that correlated with a modified water swallow test (r = −0.468, *p* < 0.0001). The study cohort was however predominantly neurological in nature (57% stroke), whereas the 4QT cohort were predominantly frail [30]. Schrock et al. (2011) conducted a similar study, based in the emergency department. Their swallow screen consisted of five questions which were aimed at the assessor not the patient. Furthermore, their cohort was predominantly stroke patients [31]. Their conclusion was that the screen was probably useful. A subsequent publication indicated that the incidence of pneumonia had been reduced when the screen was introduced [24]. The advantage of the 4QT is that it is easy to use and can be undertaken by any member of staff without prior training. There is the added advantage that in clinically busy settings and out of hours, there is no need to look for a teaspoon or to establish the time taken to swallow a fixed volume of water.

With a high NPV, it can reasonably be assumed that a person with a score of zero will not have a swallowing problem. The disadvantage is that too many people may be identified as having an impaired swallow, when this might not be the case.

## 5. Limitations

The first stage of the study can be criticized as it was not a full systematic review and—as a consequence—a systematic review protocol was not followed, and publications may have been overlooked. The 4QT study has limitations in that the cohort was small and 62.5% had a frailty score of 5 or less and were therefore only mildly frail. More than one SLT undertook the SLT screens which may have added variability to the assessment results. 

Videofluoroscopy was not undertaken in this study; however, the purpose of a swallow screen is not to diagnose the presence of aspiration, but to identify those with swallowing problems who require further assessment and possible investigation [32]. 

The 4QT asks the patient a number of questions with the answer yes/no. The screen and the person using the screen is not expected to interpret the answers, nor make a value judgement as to the nature of a change in voice after swallowing or what is a longer time taken to eat a meal.

## 6. Conclusions

This is the first study in the development of the 4QT. The 4QT would appear to be a useful, quick screening tool that can be used with no specific training. Ongoing studies are under way to further assess and validate the 4QT. 

## 7. Future Direction

Further work needs to be undertaken to validate this work, in different cohorts and with different staff groups.

Future analysis should look at whether the cut off of >0 is an all or none, whether higher scores on the 4QT are associated with more severe swallowing problems or are more predictive of a swallowing problem.

## Figures and Tables

**Table 1:** Swallow Screens/Assessments examined.

Swallow Screen/Assessment	Reference
VVST: Volume Viscosity Swallowing Test	Clave P, Arreola V, Romea M, Medina L, Palomera E, Serra-Prat M. Clin Nutr 2008; 27:806–815.
TOR-BSST (Toronto Bedside Swallowing Screening Test)	Martino R, Silver F, Teasell, Bayley M. Nicholson G, Streiner DL, Diament NE. The Toronto bedside Swallowing screening test (TOR-BSST) Stroke 2009; 40:555–561.
3-oz WST: 3-oz Water Swallow Test	DePippo KL, Holas MA, Reding ML. Arch Neurol 1992; 49:1259–1261.
Cough test	Sato M, Tohara H, Iida T, Wada S, Inoue M, Ueda K. Simplified cough test for screening silent aspiration. Arch Phys Med and Rehabilitation 2012; 93:1982–1986
BDST (Burke Dysphagia Screening Test)	DePippo KL, Holas MA, Reding MJ. Arch Phys Med Rehabil 1994;75:1284–1286
BSA: Bedside Swallowing Assessment	Smithard DG, O’Neill PA, Park C, England R, Renwick DS, Wyatt R, Morris J, Martin DF. Age and Ageing 1998; 27:99–106.
Oximetry: Oximetry	Sherman B, Niseboum JM, Jesberger BL, Morrow CA, Jesberger JA. Dysphagia 1999; 14:152–156.
PAC-SAC: Prefeeding assessment checklist-Swallowing assessment checklist	Shanley C. J Geront Nursing 2000; 26:35–48
TWST: Timed Water Swallowing Test	Nathadwarawala KM, Nicklin J, Wiles CM. JNNP 19992; 55:822–825
SBST: Simple Bedside Swallowing Test	Sitoh YY, Lee A, Phua SY, Lieu PK, Chan SP. Singapore Med J 2000; 41:376–381
BSA+O2 saturation: Combination of BSA and Oxygen Saturation Monitoring	Smith HA, Lee SH, O’Neill PA, Connolly MJ. Age and Ageing 2000; 29:495–499
DSQ: Dysphagia Screening Questionnaire	Kawashima k, Motohashi Y, Fujishima I. Dysphagia 2004; 19:266–271.
GUSS: Gugging Swallowing Screen	Trapl M, Enderle P, Nowotny M, Teuschi Y, Matz K, Dachenhausen A, Brainin M. Stroke 2007; 38:2948–2952
WSD: Westergen’s Screening for dysphagia	Westergen A, Hallberg IR, Ohlsson O. Scand J Caring Sci 1999; 13:274–282
STS-SPT: Simple Two-Step Swallowing Provocation Test	Teramoto S, Matsuse T, Fukuchi Y, Ouchi Y. Lancet 1999; 353;1243
MASA: Mann Assessment of Swallowing Ability	MASA: The Mann Assessment of Swallowing Ability. Mann G. Singular 2002.
SSA: Standardized Swallowing Assessment	Perry L. J Clin Nurs 2001; 10:463–473
CFS-D: Clinical Functional Scale for Dysphagia	Paik Nj, Soo KI, Hwan K, Oh BM, Han TR. J Korean Acad Rehab Med. 2005; 29:43–49.
Massey BSS: The Massey Bedside Swallowing Screen	Massey R, Jedicka D. J Neurosci Nurs 2002; 34:257–260.
3 non-VFG: Three non-VFG Test (Water+food test+ X ray)	Tohara H, Saitoh E, Mays KA, Kuhlemeier K, Palmer JB. Dysphagia 2003; 18:126–134.
MISA: McGill Ingestive Skills Assessment	Lambert HC, Gisel EG, Groher ME, Wood-Dauphinee S. Dysphagia 2003; 18:101–113.
DRACE: Dysphagia Risk Assessment for the Community Dwelling Elderly	Miura H, Kariyasu M, Yamasaki K, Arai Y. J Oral Rehabil 2007; 34:422–427
SSA with water/pudding: Standardized Swallowing Assessment with water/pudding	Marques CHD, de Rosso AL, Andre C. Topics in Stroke Rehabil 2008; 15:378–383.
MWST+cough test: Modified Water Swallowing Test+Cough Test	Wakasugi Y, Tohara H, Hattori F, Motohashi Y, Nakane A, Goto S, Ouchi Y. Dysphagia 2008; 23:364–370.
Emergency Dept Dysphagia Screen	Schrock JW Lou L, Ball BAW, Van Etten J. Am J Emerg Med. 2018; 36(12):2152–2154
NDST: Nursing Dysphagia Screening Tool	Bravata DM, Daggett VS, Woodward –Hagg H, Damush TM, Plue L, Russell S, Allen G, Williams LS, Hareziak J, Chumbler NR. J Rehabil Res Dev 2009; 46:1127–1134
MEOF-II: Minimal Eating Observation Form II	Westergren A, Lindholm C, Mattsson A, Ulander K. J Nutr Health Aging 2009; 13:6–12
ASDS: Acute -Stroke Dysphagia Screen	Edmiaston J, Connor LT, Loehr L, Nassief A. Am J Crit Care 2010; 19:357–364
MMASA: Modified Mann Assessment of Swallowing Ability	Antonios N, Carnaby-Mann G, Crary M, Miller L, Hubbard H, Hood K, Sambandam R, Xavier A, Silliman S. J Stroke Cerebrovasc Dis. 2010; 19:49–57.
9- indicators: 9-Clinical Indicators of Dysphagia	Boczko F. J Am Med Dir Ass 2006; 7:587–590

**Table 2:** Elements of swallow assessment/screens occurring most frequently.

Element	Frequency
Coughing with swallowing	24
Choking on swallowing	23
Voice Change with swallowing	22
Difficulty Swallowing	11
Time taken to swallow	5
Change of diet/food eaten	6
